# Housing Reconstruction in Disaster Recovery: A Study of Fishing Communities Post-Tsunami in Chennai, India

**DOI:** 10.1371/currents.dis.a4f34a96cb91aaffacd36f5ce7476a36

**Published:** 2013-04-03

**Authors:** Emmanuel Raju

**Affiliations:** Training Regions Research Centre/ Lund University Centre for Risk Assessment and Management

## Abstract

Disaster recovery after the Indian Ocean tsunami in 2004 led to a number of challenges and raised issues concerning land rights and housing reconstruction in the affected countries. This paper discusses the resistance to relocation of fishing communities in Chennai, India. Qualitative research methods were used to describe complexities in the debate between the state and the community regarding relocation, and the paper draws attention to the dimensions of the state–community interface in the recovery process. The results of this study highlight the effects of differences in the values held by each of the stakeholders regarding relocation, the lack of community participation, and thereby the interfaces that emerge between the state and the community regarding relocation. The failure to establish a nexus between disaster recovery and the importance of a sustainable livelihood for fishing communities severely delayed housing reconstruction.

## Introduction

India was one of the worst affected countries in the Indian Ocean tsunami of 2004. The tsunami not only attracted international attention, but also a considerable influx of international response and funding 1. In the process of relief and recovery, humanitarian organizations faced multiple challenges due to the scale of the disaster. According to many writers, recovery in an underdeveloped country provides an opportunity for development 2 ^,^ 3 ^,^ 4 ^,^ 5. However, recovery is considered to be the less researched areas of disaster risk management 6. In the tsunami recovery, one of the sectors facing serious problems was housing reconstruction. Four years after the tsunami, the state and the communities in Chennai were still debating and negotiating the location for reconstruction. This paper explores the discussions concerning the relocation and sustainable livelihood of fishing communities in that area.

This study is based on primary data collected in 2008, when the members of fishing communities in Srinivasapuram in Chennai were still living in damaged buildings or temporary shelters built by community members. Fifty-four members in Srinivasapuram in Chennai died in the tsunami. The community has been presented with a proposal for relocation to Semmenchery, approximately 22 km from the present location, in Kancheepuram, a neighboring district to Chennai. Furthermore, the proposed relocation site is more than a kilometer from the coast. The fishing community expressed strong resistance to relocation at a new site that was considered to be not only very far from the present location, but also too far from the coast. In this context, the aim of the present paper is to describe the arguments of the community in resisting housing relocation by answering the following research question:

What arguments do the tsunami-affected fishing communities and government authorities in Chennai express regarding housing relocation as part of tsunami recovery? 

## Disaster Recovery and Relocation: Issues and Challenges

Disaster recovery is considered to be one of the least understood aspects of disaster risk management 6 ^,^ 7 .In the present study recovery is seen as “a differential process of restoring, rebuilding and reshaping the physical, social, economic and natural environment through pre-event planning and post-event actions” 6. This definition emphasizes that recovery is a process shaped by several conditions before and after the disaster. During recovery, the built environment must be closely linked to the social aspects of a community. Although physical reconstruction is an important component of the process, it is not the only one 6 ^,^ 8 as recovery is also a social process 8 ^,^ 9 . It is identified that one of the reasons for the many definitions of disaster recovery as being the interdisciplinary nature of disaster research 10 . The recovery definition has taken a different route from its earlier conceptions of being linear 11 to being complex, multi-dimensional and non-linear process 11 ^,^ 12 ^,^ 13 . Disaster recovery must, therefore, include aspects of governance; the social aspects together with the physical aspects of housing and infrastructure as they are mutually interdependent 3 ^,^ 8 .

Great importance is attached to the cultural values of a community and its continuity during post-disaster reconstruction or relocation. Oliver-Smith 3 writes that the relocation of communities depends on their will to move to a new environment, which depends on several factors related to political, socio-cultural and economic dimensions in society. During this period, relocation can be an instigator of social conflict in the region 2 . It is not only costly to relocate an entire community, but it also changes the entire social pattern of the community 2 . Resistance to relocation also arises because of fear of loss of “social and cultural identities” 14 . Better results are obtained when a holistic view is taken in post-disaster reconstruction, including incorporating people’s points of view in planning and implementation 3 . The participation of individuals is an important issue in effective disaster recovery. During the recovery process following the 2004 tsunami, issues concerning relocation, land rights, and housing reconstruction arose in many areas along the affected coastline. Naomi Klein’s 15 work on disaster capitalism takes up the issues of land rights and eviction in post-tsunami Sri Lanka. She notes the increasing value of land along the coastline, where private players seem to be interested in capital investments. This denies the coastal communities’ access and customary rights to the land. Fishing communities in Sri Lanka were evicted from the coast once the buffer zone had been established after the tsunami. The tsunami was thus used as a trigger or an opportunity to evict these communities. Klein’s work raises the pertinent question of whether recovery can be exploited as a window of opportunity, and in that case, whose window of opportunity.

An important factor related to disaster recovery in the present study is that of sustaining fishing livelihoods. During disaster recovery, livelihoods and housing are two sectors of prime importance among many others. According to Chambers and Conway 16  , sustainable livelihoods can be understood as “that which comprises the capabilities, assets (stores, resources, claims, and access) and activities required for a means of living: a livelihood is sustainable if it can cope with and recover from stress and shocks, maintain and enhance its capabilities and assets, and provide sustainable livelihood opportunities for the next generation; and contributes net benefits to the other livelihoods at the local and global levels in the long and short term”. The Sustainable Livelihoods Framework (SLF) places people at the center of designing a livelihood program for various outcomes. This underlines the importance of community participation in decision making with regard to livelihood. Such decisions are based on historical patterns of decision making in relation to that community’s culture. Lack of respect and consideration for a community’s social and cultural values leads to failure of many reconstruction programs 17 . Cultural considerations are important to ensure sustainability of interventions undertaken as part of post-disaster reconstruction^3^^,^ 18 . In other words, recovery includes “strengthening community institutions and organizations and infrastructures, and by diversifying livelihoods” 19 . Christoplos et al. 20 suggest the culture of prevention and rights-based approaches as a policy narrative that may promote sustainable livelihoods with bringing disaster and development closer.

## Values and Social Interfaces in Disaster Recovery

Values may be referred to as “desirable trans-situational goals, varying in importance, that serve as guiding principles in the life of a person or other social entity” 21 . For example, communities attach great importance to cultural values and their continuity in post-disaster relocation and reconstruction 3 . In the present study, this conceptualization of values may be expressed as different social entities having dissimilar priorities in disaster recovery. A study on values in disaster risk reduction has revealed that there may be substantial variation in what is considered valuable when a variety of stakeholders are involved 22 . A complex situation in which values and priorities differ may give rise to social interfaces, which are defined as “critical points of intersection between different social fields, domains or life worlds, where social discontinuities based upon differences in values, social interest and power are found. Interfaces typically occur at points where different, and often conflicting, life worlds or social fields intersect” 23 . Therefore, “...interface phenomena are often embedded in critical events that tie together a number of spatially distinct, institutionally complex and culturally distinct activities” 23 . Long characterizes key elements in an interface perspective, noting that social interfaces have a long-term impact on the community. Conflicting ideas and value systems arise from the multiplicity of actors in the process. When these systems meet, a potential platform is created for conflict or other social processes of negotiation, accommodation, and cooperation. In this study, the interface perspective was adopted to identify differences in the values of different stakeholders, as well as to highlight the complexity that arises from the arguments of multiple stakeholders involved in housing relocation and disaster recovery. As social life is complex and heterogeneous, it is important to understand the long-term implications of short-term interventions 19 .

## Study Area and Research Methodology

The Indian Ocean tsunami in 2004 affected thirteen districts along the Tamil Nadu coastline of India. The area selected for this study is Srinivasapuram in Chennai (formerly known as Madras) the capital city of the state of Tamil Nadu. According to a survey of the Tamil Nadu Slum Clearance Board (TNSCB), 769 families out of a total of 3086 families living in the area belong to the fishing community, and 1161 to the non-fishing community. This region was one of the worst affected areas in Chennai, as the TNSCB reported that 54 people died in the tsunami. The occupations of people living in Srinivasapuram vary from fishing, painting, and masonry, to laborers in the construction industry. The women are mostly engaged in domestic work in the home and neighborhood, for example, as cleaning staff in some of the nearby offices. The women of the fishing community are primarily engaged in fish vending. At the time of data collection, the fishing community and the government were debating housing relocation as part of the tsunami recovery process.

The study is based on qualitative research methods. The primary data were obtained through interviews conducted in Srinivasapuram in Chennai in May 2008. In total, thirty-five respondents representing various stakeholders were interviewed in the study. The respondents consisted members of fisherfolk and non-fisherfolk in the selected area, representatives of non-governmental organizations (NGOs), and state officials who were involved in the post-tsunami recovery process in Chennai. The respondent sample consisted of twenty members from the fishing community (including men and women), six respondents from members of non-fishing communities, six respondents from NGOs and the United Nations Team for Recovery Support (UNTRS), and three respondents from government agencies.

Two rounds of in-depth interviews were conducted with the majority of the respondents in the community (members of both fishing and non-fishing communities). The interviews lasted on average for forty minutes. Purposive-snowball sampling was used to identify respondents. Respondents were identified based on their ability to provide data regarding different issues concerning the subject of the study. The respondents were also selected so as to include representatives from the local fishing governance mechanism, and respondents from fishing and other occupations. They were also identified based on their ability to provide information on the present status of recovery and relocation. Community members were also identified from different geographic locations within Srinivasapuram to ensure a holistic understanding of the process.

In addition to the interviews, two 1-hour focus group discussions were conducted with the community respondents. The first one was conducted with six young men from the fishing community, aged 18-24, involved in fishing and associated activities. The participants in this group were identified by two criteria. One was livelihood from fishing and the other was representation of different geographical parts of the study area. The other focus group discussion was conducted with eight members of the community (men and women from the fishing community). This group was identified during the interviews, based on their common views of the recovery program and housing relocation in particular. Both the focus group discussions were interactive in nature. This helped to gather multiple perspectives and issues concerning relocation and disaster recovery in the area. The interview guide was being developed as a tool for further interviews based on the initial experiences and narrations of the community. The main issues highlighted were housing relocation and problems of sustainable livelihoods. The interview guide consisted of questions about the tsunami recovery process, and the major challenges being faced in general. One of the problems often highlighted was related to housing. Further, the housing reconstruction issue was probed in particular. It raised questions leading to livelihoods and the debates between the state and the community. Later, questions included the status of housing reconstruction, the relocation details from the government, community participation, and willingness to relocate.

Semi-structured interviews were conducted with the respondents from the NGOs and the government agencies. Notes were taken during the interviews. These notes were discussed with the respondents after the interview to understand the details in accordance with the respondents. Several NGOs were working in this area, some of which had been present before the tsunami. Staff from four organizations that continued to work in the field at the time of data collection were interviewed. These organizations were identified based on their role and involvement in the housing program. Two interviews were also conducted with officials from the UNTRS, a UN coordination body for tsunami recovery activities in Tamil Nadu. The public relations officer of the Emergency Tsunami Reconstruction Project, the government project responsible for tsunami related activities, was interviewed. The Tamil Nadu Slum Clearance Board (TNSCB) is the agency responsible for tsunami housing reconstruction. Two officials from this agency who were involved in the housing relocation and reconstruction project were interviewed. These government respondents were identified as they were involved in the specific projects of housing reconstruction and also had an understanding of the tsunami recovery process in general.

The analysis of the data was inspired by grounded theory 24 . Data analysis began with the initial data collection 25 . This guided the research towards highlighting the issue of housing reconstruction as one of the most important concerns post-tsunami. During the analysis of data from the initial interviews, open coding helped to identify the different concepts and issues emerging. The data were broken down into many concepts. Axial coding was then used to identify the relationships between these concepts and to group them into broader categories. These categories helped in filtering the initial codes formed to give coherence to the data. For example, several concepts such as nature, signals, relocation, and values contributed to the category denoted Conflict of Values. Three such broad categories contributed to the findings of this study. They are: (1) Conflict of Values, (2) Community Participation in Decision Making, and (3) Transition of Debates.

## Empirical Findings

The findings of the study are presented in terms of the three broad categories emerging from the data mentioned above. The debate concerning housing relocation in Chennai was still in progress four years after the tsunami. As mentioned in the introduction, it had been proposed that the fishing communities in Srinivasapuram be relocated to a new site about 22 kilometers away. There has been strong resistance from the community to this proposal. At the time of data collection, the tsunami-affected communities had either constructed their own temporary shelters or moved into damaged houses while awaiting information on where and when permanent houses would be constructed.

**1.** **Conflict of Values**

The majority of the fisher-folk expressed their “belongingness to the sea” as an important cultural aspect of their life. These respondents stated that it would be extremely difficult to plan for fishing activities if they lived away from the coast. They also emphasized the value they place on the sea and the coast as being superior to any other form of security. One of the respondents said: “The new location is both far away from the present location and also far away from the coast”. Another respondent said: “We use signs from the sea and the sky when deciding whether to venture out on a fishing trip”; while another fisherman said: “It will be difficult to make decisions about fishing living away from the coast”. Respondents from the fishing community value their access to open space around their houses and having customary rights to the shore. They stated problems regarding space and distance to the coast at the suggested relocation site. Their daily activities are dependent on the seashore. The fishermen explained that parking of boats and drying of nets are important parts of their activities, and expressed fear that relocation to the new site may lead to theft of boats and fishing gear as they have to be left close to the shore, away from their dwellings. Women expressed concern over the lack of space for sorting, drying and even selling fish on the shore. They explained that these activities would be extremely difficult if they had to move to a new housing location more than five hundred meters from the coast. Other causes of concern expressed by the majority of the respondents were loss of access to the coast resulting from increasing tourism and privatization of coastal areas. The government respondents, however, expressed concern about damage to housing and infrastructure in future disasters. According to them, their task is to protect these communities from future disasters by rebuilding further away from the coast. The fishermen explain that living close to the sea has been a matter of livelihood sustenance. Although the government raises the importance of protecting physical infrastructure such as housing and also to protect loss of human lives, there seem to be a conflict for the two parties to come to a common agreement. Differing values of different stakeholders raise conflicts in the reconstrutcion and relocation program.

**2.** **Community Participation in Decision Making**

The majority of the respondents perceived their role in decision making during disaster recovery as minimal. They stated that they received no information about housing reconstruction and relocation for a long time after the tsunami. In one of the focus group discussions, the respondents revealed that they did not hear about the construction of new houses at a new site until early 2007, three years after the tsunami. The respondents said that their participation in determining the type of housing or design was non-existent. One of the respondents said: “We are still not sure where our new houses will be constructed”. Another respondent noted that: “After we heard about the plan for relocation, we resisted. At the moment, there is no further information about our housing”. The role of the community has since been to resist relocation. Respondents from the fishing communities and NGOs explain that the tsunami has been used as a trigger to relocate these communities away from the coast on the pretext of disaster recovery and safety. Many families whose houses were damaged returned to the shore and made use of whatever was left after the tsunami to construct their own temporary shelters, indicating that they have lost faith in the state.

The three respondents from the government agencies share a common view. They highlight the advantages of physical safety from natural disasters by relocating the community in a new area further away from the coast. When asked about community participation and issues raised by the communities, a common answer implied that the fears of the community were mere perceptions. However, these respondents agreed that the proposal for relocation was presented to the community as a formal plan, without any opportunity for consultation with or participation by the community. The majority of the respondents from the NGOs were of the opinion that the proposed relocation was a strategy for evicting these communities from the coast. Furthermore, the NGO representatives expressed the need for more community involvement in decisions that affect the livelihoods of the majority of the community. One of the NGO respondents also highlighted two major issues related to the delay in the process of arriving at a consensus or a final decision. According to him, the delay is a result of the absence of community involvement and issues of space constraints in Chennai.

**3.** **The Transition of Debates**

Respondents from the community and the NGOs highlighted that there has been a demand by the fisher-folk from the time the recovery process began to construct permanent shelters at the same location. However, there has been no information from the government to the community concerning the status of housing, the exact details of the plan, or the structure and design of the houses. As stated earlier, in reality, the state had not involved the community in decisions on their needs and what suited them best for a sustainable livelihood. The elders in the community explained that various local fishing community networks had united to voice their opposition to the proposed relocation. Respondents from the community said that one of the reasons that kept the debate and resistance to relocation alive was the strong network of fishing communities along the coast. Immediate resistance from the community caused conflicts between the two stakeholders, but the conflict with the state had evolved from resistance to negotiation over a period of approximately a year, due to the bargaining capacity of the fishing community network. The respondents from the community also say that the negotiations have not led to any consensus on rebuilding at the same location. The fishing community expressed its opposition to relocating to the proposed site as the distance to the sea would affect their livelihood. The NGOs describe the conflicts as being the result of a lack of cultural understanding in recovery interventions, and underline the importance of understanding interdependencies for sustainable recovery. The government respondents say that the community is not willing to move to a safer location. According to the government respondents, human safety and the safety of property and infrastructure are of prime importance.

## Discussion

This paper aims to map out the debate and discussion between the state and community regarding housing reconstruction and relocation of fishing communities. The results of this study highlight the effects of differences in the values held by each of the stakeholders regarding relocation, the lack of community participation, and thereby interfaces emerge between the state and the community regarding relocation. The three broad categories of findings presented above are interconnected, contributing to a delay in housing reconstruction due to resistance to relocation from the community. The conflict of values arises from the question of ‘what is valuable to whom’. According to the fishing community, the natural habitat, i.e. living close to the sea, is of prime importance. In contrast, the guiding principle of the state is to offer protection to these communities and reduce future damage. The perceptions of ‘what it is important to protect’ differ between these stakeholders. The state argues that physical safety can be ensured by building new houses away from the coast. The fisher-folk respond that there is no guarantee that fishing will provide a sustainable livelihood if they live more than five hundred meters from the coast.

According to the SLF, the integration and interaction of resources make livelihoods sustainable. The findings of this study highlight the importance of the natural environment and the ‘belongingness’ of fishing communities to the sea in daily life. They believe they have the right to access to the seashore. The debates on relocation have shown that changes in physical capital (in this case, housing) can cause serious disruption in social capital (networks), distancing the community from their natural capital (the coastline), which may have an impact on their livelihood 26 . The community must consider proximity to the coast and the design of their housing, and the connection between the two, in order to be able to make decisions that affect their livelihood. Another reason for resistance is the absence of alternative livelihoods. It is clear from the data analysis that the poor households in the research area are vulnerable because they depend heavily on the sea as their only resource, and opportunities for alternative sources of income are very limited. Disaster recovery can provide the opportunity for interventions to provide alternate strategies and enhance diversification of livelihoods. The mutual interdependence of various capitals for sustainable livelihood highlighted above goes a long way to providing a strong asset base. Lack of cultural compatibility is certainly a key issue, resulting in increasing vulnerability during post-disaster reconstruction. The cultural values of the fishing community and the regulations of the state give rise to conflicts and a clash of value systems.

The conflict between the state and the community is the central argument of this paper. Further, the two parties have not been able to make any common agreement. The community also emphasizes that it is important to provide alternate livelihood resources and build capacities to that extent. They raise concerns that lack of formal education may not provide them job opportunities outside the fishing sector. These nuances and interdependencies between various sectors such as housing and livelihoods further need to be explored and discussed when designing disaster recovery. In this process, the drive for alternate livelihoods and diversification of livelihoods may also contribute to reducing vulnerability of the fishing communities. A site for their housing reconstruction can further be explored and discussed along with these options. Since the beginning of the recovery process there has been a constant debate for the construction of permanent shelters at the original location. In the view of the state, recovery means the construction of permanent shelters and physical infrastructure. This led to conflict between the state and the community. The absence of community **consultation and participation, and the lack of involvement of the affected community **in decision making together with the lack of information about the reconstruction process exacerbated the problem. Social interfaces emerged between the state and the community, due to the clash of the formalized policy frameworks of the state and the informal fishing governance systems. The immense resistance from the community arises from an absence of dialogue between the state and the community for a long time. This communication gap was worsened when the community was not involved in the recovery process.

It is important to understand and analyse the inherent social processes of a community and its cultural values in relation to external interventions. In the present study, the problems of differing values, uncertainty regarding the possibility of a sustainable livelihood, and access to natural resources after relocation caused the emergence of social interfaces. The analysis of the data in this study reveals the conflicting relationships between the different actors. This is determined by past experience, the lack of an understanding of the cultural values of the community by the state, and the level of acceptance of the policies proposed by the state by the community. In this case, the study emphasizes on the instrumental values (such as serving the purpose of means to an end). The study further reiterates that values are difficult to measure 27 and "what individuals express as valuable in any govern situation is socially constructed" 28 . This conflict of values gives rise to interfaces due "institutionally complex and culturally distinct activities" (Long, 2001, p.84) by the community and the state. This also emphasizes that different stakeholders having varied objectives such as protecting infrastructure and lives by the state and the community emphasising to protect livelihoods by living close to the sea contributes to value conflict. Therefore, "livelihoods are both individually and jointly constructed and represent patterns of interdependencies between the needs, interests and values of particular sets of individuals or groups" 23.

Multiple discourses arise during recovery as a result of the number of actors involved in the process. With the multiplicity of actors in the process, conflicting ideas and value systems are brought together. When these systems meet, a potential platform is created for conflict or other social processes of negotiation, accommodation and cooperation. The interfaces of conflict that emerged between the state and the community were found to be important in the debate concerning reconstruction. The present study re-emphasizes the fact that livelihoods are based on interdependent factors 26 . The fishing community highlighted the nexus between the factors of physical housing reconstruction/relocation and the need to live close to the coast to make appropriate decisions regarding their livelihood. The fishing community has strong views about relocation and their customary rights to the coast in the light of coastline beautification and privatization. These conflicts regarding values and lack of community participation explain the emergence of interfaces between the stakeholders.

The data reveal a lack of community involvement in the choice of housing site and house design, which has given rise to conflict and resistance to relocation. However, the interfaces between these stakeholders have had different forms, changing from conflict to negotiation. Negotiation has come about as, in the words of Putnam 29, “A strong network can achieve shared objectives”. A solution to the interfaces between the state’s concern for physical safety and the community’s dilemma regarding a sustainable livelihood requires further dialogue, community participation along with a nuanced understanding of local culture and contexts.

## Conclusion

This study highlights the fact that the different stakeholders involved in relocation and housing reconstruction in the process of disaster recovery have differing opinions and values. Their efforts for rebuilding are therefore interdependent rather than being autonomous. These nuances arise out of the interdependence between various sectors such as housing and livelihood and the need for dialog between the different stakeholders to achieve an effective and holistic recovery plan. The International Recovery Platform 30 guidance notes on recovery highlight the ownership of recovery initiatives and participation among other key issues for effective recovery. The findings of this study confirm this, together with the need to map various stakeholders, and their values that influence discussions during the process, as these values determine what is important to each actor. The need to understand the values of the stakeholders involved is clear from the discussion on the conflict of ideas and cultural values. The debate was drawn out because of the conflict between safety and coastal regulation deemed important by the state, and the importance of living close to the sea for fisher-folk. Resistance to relocation and delay in housing reconstruction are exacerbated by a lack of understanding by the government of the culture and livelihood of the fishing communities.

In conclusion, one of the key concerns for effective disaster recovery is to take note of aspects related to continuity and change in different dimensions of the community. The community, its culture, and issues concerning sustainable livelihood must be placed at the center of any disaster recovery process. The findings of this case study also highlight the broader issues of governance in disaster recovery. The disaster recovery platform can be used to coordinate different activities and address issues of resilience building.

## Competing Interests

The authors have declared that no competing interests exist.
